# Biomarkers in Obesity-Related Metabolic Syndrome: Relationships Between LDL Subfractions, Lipid Profile, and Cardiometabolic Risk in a Slovak Population

**DOI:** 10.3390/metabo16080567

**Published:** 2026-08-11

**Authors:** Martina Gažarová, Petra Lenártová, Jana Žemberyová, Lucia Civáňová, Laura Hačková, Mária Kijovská, Lucia Šubová, Magdalena Górnicka

**Affiliations:** 1 Institute of Nutrition and Genomics, Faculty of Agrobiology and Food Resources, Slovak University of Agriculture in Nitra, Trieda Andreja Hlinku 2, 949 76 Nitra, Slovakia; martina.gazarova@uniag.sk (M.G.); xzemberyova@uniag.sk (J.Ž.); lucia.civanova@uniag.sk (L.C.); xhackoval@uniag.sk (L.H.); xkijovska@uniag.sk (M.K.); xsuboval@uniag.sk (L.Š.); 2 Institute of Human Nutrition Science, Department of Human Nutrition, Warsaw University of Life Science, Nowoursynowska 159 St., Bldg. 32, Room 2/2110, 02-766 Warsaw, Poland; magdalena_gornicka@sggw.edu.pl

**Keywords:** metabolic syndrome, weight status, body composition, anthropometric indices, cardiometabolic indices, lipid profile, LDL subfractions, metabolically unhealthy normal weight

## Abstract

**Background:** Metabolic syndrome (MetS) is associated with increased cardiometabolic risk, although substantial metabolic abnormalities may also occur in individuals with normal body weight. This study compared anthropometric characteristics, body composition, lipid profile, LDL subfractions, inflammatory markers, liver biomarkers, and novel cardiometabolic indices in participants with MetS according to obesity status and characterized the metabolic phenotype of normal-weight participants with MetS. **Methods:** This cross-sectional study included 188 Slovak adults. Anthropometric, biochemical, lipoprotein, inflammatory, liver function, and cardiometabolic parameters were assessed. Group differences were analyzed using the Mann–Whitney U test, and associations were evaluated by Spearman’s correlation analysis. **Results:** Compared with controls, participants with MetS had significantly greater adiposity, visceral fat accumulation, atherogenic lipid alterations, elevated inflammatory and liver biomarkers, higher blood pressure, and increased cardiometabolic indices (*p* < 0.05). Within the MetS group, overweight/obese participants exhibited greater visceral adiposity and higher liver enzyme concentrations, whereas most lipid parameters, LDL subfraction distribution, and several cardiometabolic indices were comparable between weight groups. VAI, TyG, TyG-BMI, TyG-WC, TyG-WHtR, LAP, AIP, and CMI showed strong correlations with visceral adiposity, triglycerides, atherogenic LDL subfractions, and smaller LDL particle size. **Conclusions:** Obesity worsens body composition and hepatic dysfunction in MetS but has limited impact on several metabolic and atherogenic characteristics. LDL-C concentration alone may not sufficiently reflect the true atherogenic risk. These findings support the metabolically unhealthy normal-weight phenotype and highlight the value of novel cardiometabolic indices and LDL subfraction analysis for improved cardiometabolic risk assessment.

## 1. Introduction

Metabolic syndrome (MetS) is a complex set of interrelated metabolic disorders that include visceral obesity, insulin resistance, atherogenic dyslipidemia, arterial hypertension, and chronic low-grade inflammation. Current knowledge indicates that the synergistic action of these factors significantly increases the risk of developing cardiovascular diseases, type 2 diabetes mellitus, and overall mortality [1,2,3]. The prevalence of metabolic syndrome has been increasing worldwide in recent decades, in parallel with the increasing prevalence of overweight and obesity. Its values vary significantly between regions of the world, depending on age, gender, ethnicity, lifestyle, and diagnostic criteria used. Epidemiological studies report a prevalence of approximately 32% in the United States and 24% in Europe according to the NCEP ATP III criteria, while in Asian and African countries it ranges from approximately 20% to more than 50% depending on the population evaluated and the diagnostic criteria used [4,5,6,7,8].

The pathogenesis of metabolic syndrome is extremely complex and involves the interaction of genetic predispositions and environmental factors, including excessive energy intake, sedentary lifestyle, insufficient physical activity, smoking, and chronic stress. Visceral adiposity is currently considered a key pathophysiological mechanism, playing a crucial role in the development of insulin resistance and chronic low-grade inflammation. Excessive accumulation of visceral adipose tissue leads to impaired adipokine secretion, increased insulin resistance, dysregulation of lipid metabolism, activation of inflammatory processes, and subsequent development of atherosclerosis. Given the central role of visceral adiposity in the pathogenesis of metabolic syndrome, waist circumference, either alone or in combination with elevated triglyceride concentrations as the hypertriglyceridemic waist phenotype, has emerged as a clinically useful surrogate marker of visceral fat accumulation and a robust predictor of increased cardiometabolic risk [9,10,11].

A significant component of metabolic syndrome is atherogenic dyslipidemia characterized by increased concentrations of triglycerides, very low-density lipoproteins (VLDL), apolipoprotein B, and reduced concentrations of HDL-C. Insulin resistance promotes the overproduction of VLDL in the liver and the formation of small dense LDL particles (sdLDL), which have a higher atherogenic potential than classic LDL particles and represent a significant predictor of atherosclerotic and cardiovascular events. Compared with larger, more buoyant LDL particles, sdLDL exhibit greater atherogenicity owing to their enhanced ability to penetrate the arterial intima, prolonged plasma residence time resulting from reduced affinity for LDL receptors, increased susceptibility to oxidative modification and glycation, and stronger binding to arterial wall proteoglycans. These properties promote foam cell formation, accelerate atherosclerotic plaque development, and make sdLDL an important predictor of atherosclerotic cardiovascular events [12,13,14,15].

Although metabolic syndrome is traditionally associated with obesity, a growing body of evidence indicates the existence of metabolically at-risk individuals with normal body weight. It is estimated that approximately one-third of individuals with a normal BMI may exhibit metabolic abnormalities characteristic of metabolic syndrome, with the distribution of adipose tissue, especially the amount of visceral fat, playing a decisive role, rather than body weight itself [16]. These individuals often remain undiagnosed, despite an increased risk of developing cardiometabolic complications. In recent years, new simple biomarkers and cardiometabolic indices have been intensively investigated for the assessment of insulin resistance, visceral obesity, metabolic dysfunction associated with steatotic liver disease (MASLD), and overall cardiometabolic risk [3,17]. The most commonly used include the triglyceride-glucose index (TyG) and its derivatives related to anthropometry [18,19,20], visceral adipose index (VAI) [21], lipid accumulation product (LAP) [22], hepatic steatosis index (HSI) [23], plasma atherogenic index (AIP) [24,25], and cardiometabolic index (CMI) [26]. These indicators represent simple and cost-effective tools that can be used to identify individuals at increased risk of metabolic and cardiovascular complications. Because these indices are derived from routinely available anthropometric and biochemical measurements, they can be easily implemented in routine clinical practice without additional costs or specialized equipment. They provide complementary information on visceral adiposity, insulin resistance, atherogenic dyslipidemia, and metabolic dysfunction that may not be captured by BMI alone, making them valuable tools for cardiometabolic risk stratification, particularly in individuals with apparently normal body weight [27,28].

Despite extensive research on metabolic syndrome, it remains unclear to what extent adiposity modifies the lipid profile, LDL subfraction distribution, and novel cardiometabolic indices in patients who already meet the diagnostic criteria for metabolic syndrome. A more detailed understanding of these differences may contribute to better identification of high-risk patients and to individualization of preventive and therapeutic strategies. Although several studies have compared obese and normal-weight individuals, the behavior of these biomarkers, the distribution of LDL subfractions, and other novel cardiometabolic indices in participants with metabolic syndrome without obesity remains insufficiently investigated.

Although obesity is considered the primary driver of metabolic syndrome, accumulating evidence suggests that metabolic dysfunction is not confined to individuals with excess body weight. A substantial proportion of adults with a normal body mass index exhibit insulin resistance, visceral adiposity, dyslipidemia, and chronic low-grade inflammation despite having a body weight within the conventional normal range. This phenotype, commonly referred to as metabolically unhealthy normal weight (MUHNW), is associated with an increased risk of cardiovascular disease, type 2 diabetes mellitus, MASLD, and all-cause mortality, with risks often comparable to those observed in individuals with overweight or obesity [29,30].

Despite growing recognition of the MUHNW phenotype, important knowledge gaps remain. Most previous studies have focused on comparing metabolically healthy and unhealthy phenotypes across different BMI categories or on evaluating the prevalence and clinical consequences of metabolic abnormalities among normal-weight populations [31]. In contrast, relatively few studies have specifically investigated whether obesity further modifies the metabolic profile of individuals who already meet the diagnostic criteria for metabolic syndrome [32]. Comprehensive data integrating body composition, LDL subfraction distribution, inflammatory biomarkers, liver function markers, and recently proposed cardiometabolic indices in normal-weight individuals with established MetS remain limited.

Therefore, the novelty of the present study lies in the comprehensive characterization of normal-weight individuals with metabolic syndrome and in the direct comparison of normal-weight and overweight/obese participants with MetS using detailed anthropometric assessment, body composition analysis, advanced lipoprotein profiling including LDL subfraction analysis, inflammatory and liver biomarkers, and multiple validated cardiometabolic indices. By addressing this underexplored phenotype, our study aims to improve the understanding of residual cardiometabolic risk beyond BMI and to identify markers that may facilitate the earlier recognition of high-risk normal-weight individuals in clinical practice.

## 2. Materials and Methods

### 2.1. Study Design and Participants

This cross-sectional observational study included 188 adult participants recruited from the Slovak population during the study period (2023–2025). From the original set of 212 volunteers, 24 individuals were excluded based on the exclusion criteria. Exclusion criteria included age <18 years, diagnosed or treated serious infectious or non-infectious diseases, pregnancy or suspected pregnancy, participation in professional sports, and contraindications to bioimpedance measurement, including vigorous physical activity immediately before measurement, unintentional or intentional weight loss of ≥5% within the previous 3 months, consumption of coffee, alcohol, or high-fat meals within 8 h before testing, and use of diuretics within 7 days before assessment.

All participants voluntarily agreed to participate and signed written informed consent before enrollment. The study protocol was approved by the Ethics Committee of the Specialized Hospital of St. Zoerardus Zobor in Nitra, Slovakia (Protocol No. 20230512/2) and was conducted in accordance with the ethical principles of the Declaration of Helsinki.

Participants were initially divided into two groups according to the presence of metabolic syndrome (MetS): the MetS group (*n* = 85) and a control group without MetS (*n* = 103). In the second stage of the analysis, only participants diagnosed with metabolic syndrome were included and further classified according to nutritional status into a subgroup with normal body weight (*n* = 21) and a subgroup with overweight or obesity (*n* = 64).

### 2.2. Definition of Metabolic Syndrome

Metabolic syndrome was diagnosed according to the harmonized criteria proposed by the Joint Interim Statement of the International Diabetes Federation (IDF), the National Heart, Lung, and Blood Institute (NHLBI), the American Heart Association (AHA), the World Heart Federation (WHF), the International Atherosclerosis Society (IAS), and the International Association for the Study of Obesity (IASO) [27].

Participants were classified as having metabolic syndrome when at least three of the following five criteria were present: elevated waist circumference according to European population-specific cut-off values (≥102 cm for men and ≥88 cm for women); triglycerides ≥ 1.7 mmol/L or treatment for hypertriglyceridemia; HDL-C < 1.0 mmol/L in men or <1.25 mmol/L in women or treatment for reduced HDL cholesterol; systolic blood pressure ≥ 130 mmHg and/or diastolic blood pressure ≥ 85 mmHg or antihypertensive treatment; fasting plasma glucose ≥ 5.6 mmol/L or previously diagnosed type 2 diabetes mellitus or antidiabetic treatment [33].

### 2.3. Anthropometric and Body Composition Assessment

Anthropometric and body composition measurements were performed using multi-frequency bioelectrical impedance analysis (MF-BIA) with the InBody 970 body composition analyser (Biospace Co., Ltd., Seoul, Republic of Korea). The instrument measures electrical impedance of five body segments at multiple frequencies (1, 5, 50, 250 and 500 kHz; 1, 2 and 3 MHz), enabling comprehensive assessment of body composition. All measurements were performed under standardized laboratory conditions by the same trained investigator to minimize inter-observer variability. Participants were examined in the morning after an overnight fast, having emptied their bladder, barefoot and wearing light clothing. Before the examination, participants were instructed to avoid alcohol consumption for 24 h, vigorous physical activity for at least 12 h, excessive food intake, and large fluid consumption before the examination. Body height was measured using the integrated electronic stadiometer (BSM370; InBody Co., Ltd., Seoul, Republic of Korea), while body weight and body composition were obtained using the InBody 970 analyser. The following anthropometric and body composition parameters were assessed: basal metabolic rate (BMR, kcal), body weight (BW, kg), body height (BH, cm), waist circumference (WC, cm), hip circumference (HC, cm), waist-to-hip ratio (WHR), waist-to-height ratio (WHtR), body mass index (BMI, kg/m^2^), fat-free mass (FFM, kg and %), skeletal muscle mass (SMM, kg and %), body fat mass (BFM, kg and %), visceral fat area (VFA, cm^2^), total body water (TBW, L), TBW/BW (%), TBW/FFM (%), extracellular water (ECW, L), ECW/TBW (%), intracellular water (ICW, L) and ICW/TBW (%).

Body mass index (BMI), waist-to-hip ratio (WHR) and waist-to-height ratio (WHtR) were calculated according to standard equations [34]. Blood pressure was measured after at least five minutes of seated rest using an automated sphygmomanometer, OMRON M7 Intelli IT with AFIB (OMRON Healthcare Co., Ltd., Shiokoji Horikawa, Shimogyoku, Kyoto, Japan) with an accuracy of ±3 mmHg of the measured value. The resulting average value of three measurements was used for the purposes of the study.

### 2.4. Laboratory Analyses

Venous blood samples were collected in the morning after a minimum of 8 h overnight fasting according to standard laboratory procedures. Whole blood samples were processed by centrifugation using a Hettich MIKRO 220R centrifuge (12-place, 35° angle rotor): EDTA tubes were centrifuged at 373 × g for 15 min, and serum gel tubes at 1036× *g* for 10 min, both at 4 °C (Andreas Hettich GmbH & Co., Tuttlingen, Germany). Separated serum and plasma samples were subsequently stored at −80 °C until further analysis. The following biochemical parameters were analyzed: fasting plasma glucose (GLU), triglycerides (TG), total cholesterol (T-C), high-density lipoprotein cholesterol (HDL-C), low-density lipoprotein cholesterol (LDL-C), very-low-density lipoprotein cholesterol (VLDL), intermediate-density lipoprotein subfractions (IDL-A, IDL-B and IDL-C), LDL subfractions (LDL-1–7), mean LDL particle size, C-reactive protein (CRP), uric acid (UA), alanine aminotransferase (ALT), alkaline phosphatase (ALP), gamma-glutamyl transferase (GGT) and aspartate aminotransferase (AST). Analyses were performed using the Biolis 24i Premium (Tokyo Boeki Machinery, Tokyo, Japan) with commercial reagents supplied by DiaSys Diagnostic Systems GmbH (Alte Strasse 9, 65558 Holzheim, Holzheim, Germany) and Randox Laboratories Ltd. (55 Diamond Road, Crumlin, County Antrim, BT29 4QY, Crumlin, United Kingdom).

Lipoprotein subfractions, including VLDL, IDL (A, B, C), and LDL (LDL-1–7), were determined in serum using the Lipoprint System LDL Subfractions Kit (Quantimetrix Corp., Redondo Beach, CA, USA) in combination with the Lipoprint^®^ analyzer, following the manufacturer’s protocol. This method is based on linear electrophoresis in non-denaturing polyacrylamide gel, enabling separation and quantification of lipoprotein subfractions. Based on LDL particle size, phenotypes were classified as non-atherogenic phenotype A (>26.8 nm), intermediate phenotype AB (26.53–26.79 nm), and atherogenic phenotype B (<26.5 nm) [35]. All Lipoprint LDL analyses were performed according to the manufacturer’s instructions under standardized laboratory conditions using calibrated equipment and identical analytical procedures throughout the study. Quality control procedures included regular instrument calibration, verification of electrophoretic separation quality, and evaluation of sample integrity before analysis. The Lipoprint LDL System has been shown to provide good analytical reproducibility and repeatability for LDL subfraction determination in previous validation studies, supporting its suitability for clinical and research applications.

### 2.5. Calculation of Cardiometabolic Indices

To comprehensively evaluate adiposity, insulin resistance, hepatic steatosis and cardiometabolic risk, the following validated indices were calculated using published equations: Visceral Adiposity Index (VAI); Hepatic Steatosis Index (HSI); Lipid Accumulation Product (LAP); Triglyceride–Glucose Index (TyG); TyG-BMI; TyG-WC; TyG-WHtR; Atherogenic Index of Plasma (AIP); Cardiometabolic Index (CMI); Total cholesterol/HDL cholesterol ratio; LDL cholesterol/HDL cholesterol ratio; Triglycerides/HDL cholesterol ratio. All indices were calculated according to the original published formulas.

### 2.6. Statistical Analysis

Statistical analyses were performed using STATISTICA 13 (TIBCO Software Inc., Palo Alto, CA, USA) and MedCalc ver.23.4.8 (MedCalc Software Ltd., Ostend, Belgium). The normality of variable distribution was assessed using the Shapiro–Wilk test. As most variables were not normally distributed, continuous data are presented as medians and interquartile ranges (IQRs). Differences between the study groups were evaluated using appropriate non-parametric statistical tests (Mann–Whitney U test). Correlations between anthropometric, biochemical and calculated cardiometabolic indices were assessed using Spearman’s rank correlation coefficient (Spearman’s rho). All statistical analyses were performed separately for the two study stages: (i) comparison of participants with and without metabolic syndrome and (ii) comparison of participants with metabolic syndrome according to weight status (normal body weight versus overweight/obesity). A two-sided *p*-value < 0.05 was considered statistically significant.

## 3. Results

### 3.1. Comparison of Anthropometric, Biochemical, and Cardiometabolic Characteristics Between Participants with and Without Metabolic Syndrome (MetS)

Table 1 compares anthropometric and body composition parameters between participants with and without metabolic syndrome (MetS). Participants with metabolic syndrome had significantly higher body weight, BMI, waist circumference, hip circumference, WHR, WHtR, body fat mass (BFM, kg and %), and visceral fat area (VFA). Conversely, they had lower relative fat-free mass (FFM %) and a lower TBW/BW ratio. Differences in age, height, basal metabolic rate, total body water, extracellular water, and ECW/TBW ratio were not statistically significant (*p* > 0.05).

Table 2 shows the comparison of biochemical, lipoprotein, inflammatory, hepatic, and hemodynamic parameters between participants with and without metabolic syndrome (MetS). In the group with MetS, there were significantly higher TG, higher VLDL, higher concentration of LDL-2, lower HDL-C, and a smaller average size of LDL particles. T-C, LDL-C, and GLU did not differ significantly (*p* > 0.05). The group with MetS also had significantly higher values of CRP, UA, and GGT. However, ALT, AST, and ALP were not significantly different (*p* > 0.05). In the case of blood pressure, participants with MetS had significantly higher systolic and diastolic pressure; heart rate did not differ significantly between groups (*p* > 0.05).

Lipid profile analysis showed that participants with metabolic syndrome had significantly higher concentrations of TG (1.20 vs. 0.83 mmol/L; *p* < 0.001) and VLDL cholesterol (1.09 vs. 0.93 mmol/L; *p* < 0.01), while HDL-C concentration was significantly lower (1.45 vs. 1.70 mmol/L; *p* < 0.001). In contrast, concentrations of T-C, LDL-C, GLU and most LDL subfractions did not differ significantly between groups (*p* > 0.05). However, a more detailed analysis of LDL subfractions revealed qualitative differences in the distribution of lipoprotein particles. Participants with metabolic syndrome had a significantly higher LDL-2 subfraction concentration (*p* < 0.001) and, at the same time, a smaller mean LDL particle size (the difference in distribution was statistically significant, *p* < 0.05). Although the median LDL size was similar in both groups, the narrower distribution of values in the group without metabolic syndrome suggests a more favorable representation of larger, less atherogenic LDL particles. In contrast, in participants with metabolic syndrome, a shift toward smaller and denser LDL particles, which have a higher atherogenic potential, was observed.

Table 3 compares metabolic, atherogenic, and cardiometabolic indices between participants with and without metabolic syndrome (MetS). All observed indices were significantly higher in participants with metabolic syndrome. All differences reached high statistical significance (*p* < 0.001).

### 3.2. Comparison of Anthropometric, Biochemical, and Cardiometabolic Characteristics Between Normal-Weight and Overweight/Obese Participants with Metabolic Syndrome (MetS)

Within the group of participants with metabolic syndrome, individuals with overweight or obesity (MetSO; *n* = 64) and participants with normal body weight (MetSnO; *n* = 21; Table 4) were compared. Neither age nor body height differed significantly between the groups, which makes it possible to interpret the observed differences primarily as a consequence of the different degrees of adiposity. The MetSO group showed significantly higher values of body weight, BMI, waist circumference, hip circumference, WHR, WHtR, amount of body fat, visceral adipose tissue area, fat-free body mass, skeletal muscle mass, and total body water (*p* < 0.001). At the same time, the values of extracellular and intracellular water were higher in this group. However, the relative representation of fat, muscle mass, or fat-free mass did not differ significantly between groups, suggesting that the difference was primarily in the absolute abundance of body compartments.

Despite significant differences in body composition, most parameters of the basic lipid profile did not differ significantly between the groups (Table 5). Concentrations of TG, HDL-C, VLDL, and GLU were comparable. Significantly higher concentrations of LDL-C were found in participants with normal body weight (*p* < 0.05). Similarly, these individuals had higher concentrations of IDL-A and LDL-1 subfractions (*p* < 0.05), while concentrations of the more atherogenic LDL-2 to LDL-7 subfractions and mean LDL particle size did not differ significantly between the groups. Significantly higher concentrations of UA, ALT, AST, and GGT were found in the MetSO group, which indicates a more significant metabolic burden on the liver and probably a higher prevalence of non-alcoholic fatty liver. An interesting finding was higher CRP values in normal-weight participants with metabolic syndrome. This result may be due to the small sample size, individual variability, or the presence of other inflammatory factors not directly related to the degree of obesity. Neither systolic nor diastolic blood pressure differed significantly between groups, suggesting that the presence of metabolic syndrome alone is the dominant determinant of elevated blood pressure regardless of body weight. However, obese participants had higher heart rates, which may reflect increased sympathetic nervous system activity.

Evaluation of cardiometabolic indices revealed significantly higher values of HSI, LAP, TyG-BMI, TyG-WC, and TyG-WHtR in the MetSO group (Table 6). In contrast, no significant differences were observed between the groups in the TyG index, VAI, AIP, CMI, or lipid ratios (TC/HDL, LDL/HDL, and TG/HDL). These findings suggest that indices incorporating anthropometric variables are more sensitive to differences in the degree of adiposity, whereas indices based primarily on metabolic parameters reflect the presence of metabolic syndrome itself regardless of body weight.

### 3.3. Spearman Correlation Analysis of Metabolic, Atherogenic, and Cardiometabolic Indices with Clinical and Biochemical Characteristics According to Weight Status in Participants with Metabolic Syndrome (MetS)

In the group of participants with metabolic syndrome and current overweight/obesity, BMI, TyG-BMI, TyG-WC and TyG-WHtR showed very strong positive correlations with body weight, waist circumference, hip circumference, WHR, body fat mass and visceral fat mass (mostly r > 0.70), which is consistent with their close relationship with the degree of adiposity (Table 7).

The metabolic indices TyG, LAP, and their combined variants were significantly correlated with glucose concentration and especially with TG, with TyG showing very strong correlations with TG (r = 0.936; Table 8). At the same time, significant positive correlations were found with the concentrations of VLDL, IDL-B, IDL-C, and atherogenic LDL subfractions, while the average LDL particle size showed significant negative correlations. The relationships between the atherogenic indices AIP, CMI, TG/HDL, LDL/HDL, and lipid parameters were also significant. These indices strongly correlated with the concentration of TG, LDL-2, LDL-3, and LDL-3–7, while simultaneously showing negative correlations with HDL-C and the average LDL particle size. This profile is characteristic of atherogenic dyslipidemia associated with insulin resistance. In the MetSO group, we also observed significant correlations with biochemical indicators of liver damage. HSI showed a strong positive correlation with ALT, while GGT was positively correlated with most of the evaluated indices. Uric acid was positively associated with most of the anthropometric and atherogenic indices, supporting its relationship to metabolic risk. CRP correlated mainly with LAP, TyG, TyG-WHtR, and other indices of central adiposity, suggesting an association between visceral obesity and chronic low-grade inflammation.

Spearman correlation analysis in participants with metabolic syndrome without obesity showed that most of the assessed adiposity and atherogenic indices were significantly correlated with anthropometric parameters. The strongest correlations were recorded between BMI, HSI, TyG-BMI, TyG-WC, and TyG-WHtR indices and body composition indicators, with correlation coefficients reaching values up to r = 0.91, indicating a very strong positive association (Table 9).

Significant positive correlations were observed between most indices and the concentrations of TG, VLDL, IDL-B, LDL-2, LDL-3, and LDL3–7 fractions (Table 10). Conversely, mean LDL particle size showed consistent negative correlations with most of the observed indices, suggesting that higher values of atherogenic indices are associated with the predominance of smaller and denser LDL particles characteristic of an atherogenic lipid profile. An interesting finding was the significant positive correlations between VAI, LAP, TyG, TyG-WC, TyG-BMI, TyG-WHtR, AIP, and CMI indices with concentrations of VLDL and atherogenic LDL subfractions, with the highest correlation coefficients being recorded for VLDL and LDL-2/LDL-3. At the same time, significant negative correlations were recorded with LDL particle size, supporting the association of these indices with atherogenic remodeling of the lipoprotein spectrum. Conversely, the relationships between individual indices and markers of inflammation, liver enzymes, and blood pressure were less consistent and in most cases did not reach statistical significance. Similarly, age did not show significant associations with most of the evaluated indices.

## 4. Discussion

The present study showed that participants with metabolic syndrome differed significantly from participants without metabolic syndrome in several anthropometric, biochemical, and cardiometabolic parameters, while the differences are consistent with the typical phenotype of metabolic syndrome. The results confirm that metabolic syndrome is closely associated mainly with central (abdominal) obesity and the accumulation of visceral fat, which represents the most metabolically active adipose tissue producing pro-inflammatory cytokines and supporting the development of insulin resistance [2,11,36]. This is a typical atherogenic dyslipidemia of metabolic syndrome characterized by hypertriglyceridemia, a decrease in HDL-C and a shift towards smaller LDL particles, which have a higher atherogenic potential [2,11]. Lipid spectrum analysis showed significantly higher concentrations of TG, VLDL, and LDL-2 subfraction in participants with metabolic syndrome, while HDL-C concentration was significantly lower. The average size of LDL particles was slightly, but statistically significantly lower in the group with metabolic syndrome. On the contrary, the concentrations of T-C, LDL-C, GLU, or other LDL subfractions did not differ significantly between the groups. Higher CRP indicates chronic low-grade inflammation characteristic of metabolic syndrome [37]. Increased UA supports the presence of insulin resistance, and higher GGT may indicate early signs of non-alcoholic fatty liver disease [37,38]. The results also confirm that arterial hypertension remains one of the basic clinical manifestations of metabolic syndrome [39]. The most significant differences were noted in the new metabolic indices (TyG, LAP, VAI, CMI, AIP and lipid ratios).

The study further compared two phenotypes of metabolic syndrome, overweight/obese individuals and metabolically unhealthy normal-weight individuals. Although obesity significantly affected body composition, visceral adiposity, and liver-related biomarkers, relatively few differences were observed in lipid profiles, LDL subfractions, and most cardiometabolic indices. These findings suggest that the presence of metabolic syndrome itself, rather than increased body weight, may be the main determinant of metabolic dysregulation. As expected, overweight or obese participants had significantly higher body weight, BMI, WC, WHR, WHtR, VFA, and BFM than normal-weight individuals with MetS. These findings are consistent with the current concept that visceral adipose tissue is a major factor in insulin resistance through increased free fatty acid flux, dysregulation of adipokines, and chronic low-grade inflammation. Increased visceral adiposity promotes ectopic lipid storage, mitochondrial dysfunction, and systemic inflammation, thereby contributing to increased cardiometabolic risk [2,40].

Despite significant differences in obesity, TG, HDL-C, VLDL, and GLU concentrations were not significantly different between the two MetS phenotypes. Similarly, TyG, VAI, AIP, CMI, and lipid ratios remained comparable. These observations suggest that once the metabolic syndrome is established, metabolic abnormalities related to insulin resistance may already be fully manifested regardless of BMI. This finding supports the concept of MUHNW individuals who exhibit metabolic abnormalities comparable to obese individuals despite having normal body weight. Such individuals have been repeatedly reported to have an increased cardiometabolic risk despite apparently normal anthropometric characteristics [40,41].

These findings are consistent with recent evidence indicating that metabolic dysfunction rather than excess body weight is the principal determinant of the atherogenic lipid phenotype once metabolic syndrome has developed. Recent reviews have emphasized that insulin resistance, visceral adipose tissue dysfunction, and ectopic fat accumulation are the key mechanisms driving hepatic very-low-density lipoprotein overproduction, impaired triglyceride metabolism, and lipoprotein remodeling, irrespective of BMI [28,42]. Consequently, individuals with normal body weight who fulfill the diagnostic criteria for MetS may exhibit lipid abnormalities comparable to those observed in overweight or obese individuals. Similar observations have been reported in studies of the metabolically unhealthy normal-weight phenotype, demonstrating that metabolic health status is a stronger determinant of cardiometabolic risk than BMI alone [43]. Therefore, the absence of significant differences in most conventional lipid parameters between our study groups is consistent with the current understanding that the metabolic consequences of insulin resistance may largely outweigh the additional contribution of obesity once MetS is established.

Analysis of LDL subfractions showed that overweight or obese individuals did not have higher concentrations of small dense LDL particles or reduced LDL particle size. Instead, normal-weight participants showed slightly higher concentrations of LDL cholesterol, LDL-1, and IDL-A, while LDL-2 to LDL-7 and mean LDL particle size remained comparable between groups. Previous studies have shown that metabolic syndrome is typically associated with a shift toward smaller, denser LDL particles, which is mainly driven by hypertriglyceridemia and insulin resistance, rather than obesity per se [13,44,45]. Our findings may therefore suggest that LDL particle remodeling had already occurred in both groups, as all participants met the diagnostic criteria for metabolic syndrome. Consequently, obesity did not further worsen the LDL subfraction profile beyond the already present metabolic abnormalities.

Higher LDL cholesterol and LDL-1 concentrations observed in normal-weight individuals should not necessarily be interpreted as a less favorable lipid profile. LDL-1 represents larger LDL particles that are considered to be significantly less atherogenic than small, dense LDL particles. Therefore, absolute LDL cholesterol concentration alone may not adequately reflect cardiovascular risk without a concomitant assessment of LDL particle characteristics. This observation further supports previous evidence that qualitative changes in LDL composition provide more clinically relevant information than conventional lipid parameter measurements alone [14,45,46]. Markers reflecting liver involvement differed substantially between groups. Overweight or obese participants showed significantly higher values of ALT, AST, GGT, UA, and HSI, indicating more pronounced metabolic liver dysfunction and a higher likelihood of MASLD. Fat accumulation in the liver represents one of the main consequences of visceral obesity and insulin resistance and significantly contributes to further cardiometabolic deterioration [17]. The significantly higher LAP indices and anthropometrically adjusted TyG indices (TyG-BMI, TyG-WC and TyG-WHtR) observed in obese participants are therefore expected, as these indices include obesity measures in their calculation and are strongly associated with visceral fat accumulation. The present findings suggest that in participants with metabolic syndrome and obesity, atherogenic indices not only reflect the degree of adiposity, but also very well characterize disorders of glucose metabolism, atherogenic dyslipidemia, unfavorable distribution of LDL subfractions, and signs of hepatic dysfunction. Correlations were systematically stronger in this group than in participants without obesity, which indicates an enhanced influence of obesity on the interconnection of metabolic risk factors.

The results of Spearman correlation analysis confirmed that the observed anthropometric, atherogenic and insulin resistance-reflecting indices represent significant markers of metabolic changes characteristic of the metabolic syndrome. At the same time, it was shown that the strength of these relationships was in most cases higher in participants with metabolic syndrome with overweight/obesity than in participants without obesity, which indicates the significant role of excessive adiposity in the progression of metabolic disorders. In the MetSO group, the indices BMI, TyG-BMI, TyG-WC, and TyG-WHtR showed very strong correlations with indicators of both total and visceral adiposity, especially with waist circumference, body fat mass, and visceral adipose tissue. These results are consistent with the knowledge that central obesity represents the main pathophysiological determinant of insulin resistance and metabolic syndrome. Visceral adipose tissue is metabolically highly active, produces pro-inflammatory cytokines (TNF-α, IL-6), reduces adiponectin secretion, and increases the influx of free fatty acids into the portal circulation, thereby promoting hepatic insulin resistance, increased VLDL synthesis, and the development of atherogenic dyslipidemia [40,47]. Chronic expansion of visceral fat also promotes ectopic lipid accumulation in skeletal muscle, which further worsens insulin resistance and cardiovascular risk [3].

Very strong correlations of the TyG index and its derivatives with TG, GLU and VLDL concentrations confirm that TyG is among the most reliable indirect markers of insulin resistance. Several meta-analyses have shown that the TyG index closely correlates with the results of the hyperinsulinemic-euglycemic clamp test and the HOMA-IR index and represents a simple, inexpensive, and reproducible marker of metabolic risk [48,49]. An important observation was the significant increase in the TyG index despite the absence of significant differences in plasma glucose between participants with metabolic syndrome and the control group. This finding strongly supports the current concept that insulin resistance develops long before fasting hyperglycemia becomes clinically apparent. Since fasting glucose remains tightly regulated by compensatory hyperinsulinemia during the early stages of insulin resistance, glucose concentrations can remain within the normal range, while triglyceride metabolism is already significantly impaired [3]. As a result, TyG appears to represent a more sensitive early biomarker of metabolic deterioration than glucose alone and may facilitate earlier identification of individuals at increased cardiometabolic risk. Even higher diagnostic value is achieved by the combined indices TyG-BMI, TyG-WC, and TyG-WHtR, which integrate insulin resistance with a measure of central adiposity [20,50]. The fact that these indices showed the strongest correlations in the MetSO group supports their potential use in identifying participants at high cardiometabolic risk.

A significant finding was the strong association of most of the monitored indices with atherogenic lipoprotein subfractions, especially with VLDL, IDL-B, LDL-2, LDL-3, and LDL-3–7, together with a simultaneous negative correlation with the average LDL particle size. This finding reflects the typical picture of atherogenic dyslipidemia characteristic of insulin resistance, in which there is increased production of VLDL in the liver, subsequent remodeling of LDL particles by cholesteryl ester transfer protein (CETP), and the formation of small dense LDL particles. It is precisely small dense LDL particles that have higher atherogenicity due to their increased oxidizability, longer circulation half-life, and greater ability to penetrate the vascular wall [1,51,52]. Therefore, negative correlations with the average LDL size can be considered a significant marker of increasing atherosclerotic risk. Small dense LDL particles are recognized as one of the hallmarks of metabolic syndrome and contribute significantly to accelerated atherosclerosis independently of traditional lipid parameters. Oxidized LDL initiates endothelial dysfunction, macrophage foam cell formation, and atherosclerotic plaque progression, making LDL subfraction analysis a valuable adjunct to cardiovascular risk assessment. These findings support the concept that advanced lipoprotein phenotyping can identify high-risk individuals who would not be recognized using conventional lipid parameters alone [14].

The strong correlations of AIP, CMI, and TG/HDL indices with atherogenic LDL subfractions support their importance as indicators of qualitative changes in the lipoprotein spectrum. Previous studies have shown significant associations between increased AIP, abdominal obesity, insulin resistance, and cardiovascular risk, findings that closely correspond to the current results [24,25]. Similarly, the CMI, which combines the TG/HDL ratio with WHtR, reflects both lipid metabolism disorders and central obesity, and several studies have documented its relationship to coronary atherosclerosis and metabolic syndrome [26,53,54].

In contrast to the MetSO group, the correlations in the MetSnO group were less numerous and generally weaker, although significant associations between atherogenic indices and lipoprotein subfractions were maintained. This result supports the current concept of the metabolically unhealthy normal-weight individual, according to which even individuals without manifest obesity can exhibit significant insulin resistance, ectopic fat accumulation, and atherogenic dyslipidemia [41,55]. The presence of significant correlations with VLDL and small dense LDL particles suggests that metabolic risk is not determined solely by BMI, but primarily by the quality of adipose tissue and body fat distribution. In addition, significant correlations were observed between HSI, ALT, and GGT in the MetSO group, supporting their relationship with MASLD. The HSI was originally developed as a simple non-invasive tool to estimate hepatic steatosis, and its significant correlations with liver enzymes in our cohort confirm its clinical utility [56]. At the same time, positive correlations of GGT with several atherogenic indices indicate a close relationship between hepatic insulin resistance, oxidative stress, and atherogenic dyslipidemia. Positive associations between uric acid and most of the monitored indices are in line with works documenting the role of hyperuricemia in the development of insulin resistance, endothelial dysfunction, and systemic inflammation [57]. Similarly, correlations of CRP with indices of central adiposity support the concept of chronic low-grade inflammation as one of the main mechanisms linking visceral obesity with the development of cardiometabolic complications.

This study has several limitations that need to be taken into account when interpreting the results. One of the main limitations of the present study is the relatively small number of normal-weight participants with metabolic syndrome compared with the overweight/obese MetS group. This imbalance reflects the lower prevalence of the metabolically unhealthy normal-weight phenotype in the general population, making recruitment of these individuals particularly challenging. The unequal sample sizes may have reduced the statistical power to detect subtle differences in some biochemical, lipid, and LDL subfraction parameters. Therefore, the absence of statistically significant differences in certain variables should be interpreted with caution, as it may partly reflect limited statistical power rather than the absence of biologically relevant differences. Nevertheless, despite the smaller sample size, the inclusion of this clinically underrecognized phenotype represents an important strength of the study, providing additional insight into the metabolic heterogeneity of individuals with metabolic syndrome across different BMI categories. At the same time, the study population consisted exclusively of Slovak adults, which may limit the generalizability of the obtained results to populations with different ethnic or geographical origins. Another limitation of the present study is its cross-sectional design, which precludes the establishment of causal relationships between obesity status, lipid abnormalities, LDL subfraction characteristics, inflammatory markers, and cardiometabolic indices. Therefore, the observed findings should be interpreted as associations rather than evidence of cause-and-effect relationships. Future prospective and longitudinal studies are needed to clarify the temporal sequence and potential causal mechanisms underlying these associations.

Despite the limitations, the strength of the study is the comprehensive assessment of participants, including anthropometric parameters, body composition analysis, inflammatory biomarkers, liver enzymes, detailed analysis of lipoprotein subfractions, and several novel cardiometabolic indices. This multidimensional approach allowed for a more detailed characterization of metabolic syndrome phenotypes and the identification of relationships that might have been missed using conventional anthropometric and lipid parameters alone.

## 5. Conclusions

This study showed that obesity in participants with metabolic syndrome is associated with unfavorable body composition characteristics, characterized by increased adiposity, higher visceral fat accumulation, and adverse liver-related metabolic markers. However, no significant differences were found between obese and non-obese participants with metabolic syndrome in most lipid profile parameters, distribution of atherogenic LDL subfractions, or multiple cardiometabolic indices. Comparable values of TyG, AIP, CMI, LAP, VAI, and TG/HDL ratios support the concept of a metabolically unhealthy normal-weight phenotype and suggest that significant metabolic dysregulation may persist even at normal body weight.

The results also highlighted the potential value of modern cardiometabolic indices, especially TyG, TyG-BMI, TyG-WC, TyG-WHtR, LAP, VAI, AIP, and CMI, as complementary markers that can better reflect metabolic disturbances than conventional biochemical indicators alone. The significant associations of these indices with atherogenic LDL subfractions, LDL particle size, and visceral adiposity indicators point to their potential to enable more accurate stratification of cardiometabolic risk. Our findings are consistent with the concept that LDL-C concentration alone may not sufficiently reflect the true atherogenic risk, and that the analysis of lipoprotein subfractions may provide additional clinically relevant information. From a clinical perspective, our findings emphasize that screening strategies should include not only indicators of visceral adiposity and insulin resistance, but also advanced parameters of lipid metabolism, which allow the identification of individuals with high residual cardiometabolic risk even before the development of manifest cardiovascular complications. Future longitudinal research should verify the prognostic significance of these indices and clarify the mechanisms of metabolic dysregulation in participants with metabolic syndrome without obesity.

## Figures and Tables

**Table 1:** Comparison of anthropometric and body composition parameters between participants with and without metabolic syndrome (MetS).

	MetS YES (*n* = 85)				MetS NO (*n* = 103)				
	Med	IQR	Min	Max	Med	IQR	Min	Max	*p*-Value
Age [years]	46.00	41.00–53.00	22.00	67.00	47.00	41.00–53.00	19.00	68.00	>0.05
BMR [kcal]	1490	1383–1909	1172	2542	1428	1350–1711	1176	2174	>0.05
BW [kg]	83.50	70.78–93.70	46.80	131.00	71.30	62.43–86.30	53.40	103.70	<0.001
BH [cm]	168.00	165.00–176.00	154.00	193.70	169.90	164.00–174.00	157.55	191.28	>0.05
WC [cm]	97.60	90.90–106.63	71.60	146.80	85.30	80.58–100.48	71.50	124.30	<0.001
HC [cm]	104.10	97.35–109.68	84.50	122.90	97.90	93.33–104.40	87.80	116.50	<0.001
WHR	0.95	0.89–1.00	0.68	1.19	0.90	0.85–0.96	0.71	1.08	<0.001
WHtR	0.57	0.53–0.63	0.39	0.84	0.50	0.47–0.58	0.39	0.78	<0.001
BMI [kg/m^2^]	28.60	25.26–31.60	16.90	42.53	24.88	22.00–28.92	16.70	40.76	<0.001
FFM [kg]	51.90	47.38–71.23	37.40	100.50	49.00	45.25–62.10	37.30	83.50	<0.05
FFM [%]	67.83	59.42–77.66	50.36	93.29	72.63	65.12–80.88	49.50	96.31	<0.05
SMM [kg]	28.70	25.84–40.76	20.10	59.50	26.90	24.75–34.83	20.00	48.50	<0.05
SMM [%]	37.85	32.71–43.42	27.76	54.59	40.03	35.88–44.38	26.92	55.34	>0.05
BFM [kg]	25.20	17.73–34.00	5.70	57.70	18.40	11.93–27.00	2.80	51.40	<0.001
BFM [%]	32.10	22.39–40.61	6.71	49.63	27.40	19.10–34.94	3.69	50.48	<0.05
VFA [cm^2^]	109.50	79.90–142.08	6.00	223.73	77.60	53.03–122.57	5.00	210.80	<0.001
TBW [L]	37.90	34.60–52.38	27.40	73.50	36.00	33.20–45.50	27.30	61.20	>0.05
TBW/BW [%]	49.64	43.45–56.94	37.05	68.35	53.19	47.62–59.33	36.40	70.49	<0.05
TBW/FFM [%]	73.30	73.18–73.47	72.84	73.62	73.33	73.19–73.49	72.84	73.76	>0.05
ECW [L]	14.50	13.28–19.48	10.50	26.40	13.90	12.60–17.03	10.40	22.70	>0.05
ECW/TBW [%]	37.85	37.36–38.42	35.92	39.60	38.10	37.55–38.49	36.12	39.38	>0.05
ICW [L]	23.60	21.35–32.80	16.90	47.10	22.10	20.53–28.25	16.90	38.70	<0.05
ICW/TBW [%]	62.15	61.58–62.65	60.41	64.08	61.91	61.51–62.45	60.62	63.88	>0.05

MetS, metabolic syndrome; Med, Median; IQR, Interquartile Range; Min, Minimum; Max, Maximum; BMR, Basal Metabolic Rate; BW, Body Weight; BH, Body Height; WC, Waist Circumference; HC, Hip Circumference; WHR, Waist-to-Hip Ratio; WHtR, Waist-to-Height Ratio; BMI, Body Mass Index; FFM, Fat-Free Mass; SMM, Skeletal Muscle Mass; BFM, Body Fat Mass; VFA, Visceral Fat Area; TBW, Total Body Water; ECW, Extracellular Water; ICW, Intracellular Water.

**Table 2:** Comparison of biochemical, lipoprotein, inflammatory, liver function, and hemodynamic parameters between participants with and without metabolic syndrome (MetS).

	MetS YES (*n* = 85)				MetS NO (*n* = 103)				
	Med	IQR	Min	Max	Med	IQR	Min	Max	*p*-Value
GLU [mmol/L]	5.20	4.70–5.63	3.00	12.69	5.20	4.70–5.55	3.22	6.20	>0.05
TG [mmol/L]	1.20	0.97–1.85	0.43	3.22	0.83	0.68–1.10	0.38	1.65	<0.001
T-C [mmol/L]	5.67	4.67–6.44	3.48	8.77	5.40	4.74–5.97	2.85	7.47	>0.05
HDL-C [mmol/L]	1.45	1.20–1.65	0.40	2.48	1.70	1.48–1.91	1.20	3.72	<0.001
LDL-C [mmol/L]	3.35	2.70–4.06	1.74	7.05	3.01	2.59–3.70	1.17	5.57	>0.05
VLDL [mmol/L]	1.09	0.88–1.30	0.49	1.73	0.93	0.76–1.16	0.57	9.15	<0.01
IDL-A [mmol/L]	0.57	0.43–0.76	0.08	1.24	0.67	0.49–0.85	0.08	1.24	<0.05
IDL-B [mmol/L]	0.41	0.30–0.54	0.10	1.29	0.39	0.28–0.52	0.08	0.83	>0.05
IDL-C [mmol/L]	0.52	0.41–0.67	0.23	1.03	0.52	0.41–0.62	0.13	0.91	>0.05
LDL-1 [mmol/L]	0.98	0.77–1.13	0.28	1.73	0.96	0.75–1.14	0.21	1.60	>0.05
LDL-2 [mmol/L]	0.47	0.21–0.71	0.05	1.32	0.31	0.19–0.44	0.00	0.96	<0.001
LDL-3 [mmol/L]	0.05	0.00–0.16	0.00	0.83	0.03	0.00–0.08	0.00	0.41	>0.05
LDL-4 [mmol/L]	0.00	0.00–0.00	0.00	0.41	0.00	0.00–0.00	0.00	0.08	>0.05
LDL3-7 [mmol/L]	0.05	0.00–0.16	0.00	1.24	0.03	0.00–0.08	0.00	0.47	>0.05
Mean LDL size [nm]	27.20	26.80–27.40	25.60	27.60	27.20	27.10–27.40	26.40	27.70	<0.05
CRP [mg/L]	4.30	1.20–5.93	0.00	25.32	3.00	0.51–4.90	0.02	10.70	<0.05
UA [μmol/L]	294.92	247.75–358.25	146.00	500.00	270.00	226.50–316.10	157.00	453.00	<0.05
ALT [μkat/L]	0.32	0.22–0.44	0.10	6.58	0.31	0.21–0.40	0.06	2.66	>0.05
ALP [μkat/L]	1.25	1.02–1.57	0.62	2.10	1.21	1.01–1.53	0.62	2.54	>0.05
GGT [μkat/L]	0.34	0.28–0.47	0.16	1.31	0.29	0.24–0.40	0.14	1.64	<0.05
AST [μkat/L]	0.36	0.30–0.43	0.20	2.88	0.35	0.30–0.42	0.18	1.58	>0.05
BPs [mmHg]	132.00	121.75–139.25	106.00	155.00	119.00	111.25–123.00	92.00	130.00	<0.001
BPd [mmHg]	84.00	75.75–90.00	51.00	102.00	77.00	70.25–82.00	53.00	98.00	<0.001
Heart rate [beat/min.]	70.00	62.00–79.25	43.00	112.00	68.00	61.00–75.00	41.00	110.00	>0.05

MetS, metabolic syndrome; Med, Median; IQR, Interquartile Range; Min, Minimum; Max, Maximum; GLU, Glucose; TG, Triglycerides; T-C, Total Cholesterol; HDL-C, High-Density Lipoprotein Cholesterol; LDL-C, Low-Density Lipoprotein Cholesterol; VLDL, Very-Low-Density Lipoprotein Cholesterol; IDL-A, Intermediate-Density Lipoprotein Subfraction A; IDL-B, Intermediate-Density Lipoprotein Subfraction B; IDL-C, Intermediate-Density Lipoprotein Subfraction C; LDL-1, Low-Density Lipoprotein Subfraction 1; LDL-2, Low-Density Lipoprotein Subfraction 2; LDL-3, Low-Density Lipoprotein Subfraction 3; LDL-4, Low-Density Lipoprotein Subfraction 4; LDL3-7, Low-Density Lipoprotein Subfractions 3–7; CRP, C-Reactive Protein; UA, Uric Acid; ALT, Alanine Aminotransferase; ALP, Alkaline Phosphatase; GGT, Gamma-Glutamyl Transferase; AST, Aspartate Aminotransferase; BPs, Systolic Blood Pressure; BPd, Diastolic Blood Pressure.

**Table 3:** Comparison of metabolic, atherogenic, and cardiometabolic indices between participants with and without metabolic syndrome (MetS).

	MetS YES (*n* = 85)				MetS NO (*n* = 103)				
	Med	IQR	Min	Max	Med	IQR	Min	Max	*p*-Value
VAI	1.64	1.14–2.42	0.51	4.40	0.84	0.64–1.30	0.24	2.48	<0.001
HSI	37.27	33.40–40.57	23.47	62.30	32.95	29.76–37.86	23.17	49.20	<0.001
LAP [cm*mmol/L]	45.83	31.85–69.87	6.84	179.70	21.35	15.20–36.89	4.29	99.45	<0.001
TyG	8.54	8.21–9.00	7.58	9.55	8.14	7.86–8.46	7.20	8.91	<0.001
TyG-BMI [kg/m^2^]	243.48	223.28–268.40	128.06	374.79	196.43	179.90–236.86	133.78	359.07	<0.001
TyG-WC [cm]	825.12	778.04–904.56	559.98	1293.67	687.64	654.55–810.30	531.59	1094.99	<0.001
TyG-WHtR	4.88	4.43–5.43	2.93	7.37	4.04	3.74–4.75	2.88	6.87	<0.001
AIP	−0.01	−0.19–0.13	−0.56	0.49	−0.31	−0.42–−0.18	−0.72	0.11	<0.001
CMI	0.54	0.37–0.78	0.12	1.69	0.24	0.19–0.33	0.09	0.86	<0.001
T-C/HDL	3.91	3.16–4.84	2.22	10.95	3.15	2.71–3.64	1.66	5.13	<0.001
LDL/HDL	2.37	1.79–3.09	0.96	8.36	1.88	1.43–2.24	0.48	3.90	<0.001
TG/HDL	0.97	0.64–1.35	0.28	3.08	0.49	0.38–0.66	0.19	1.28	<0.001

MetS, metabolic syndrome; Med, Median; IQR, Interquartile Range; Min, Minimum; Max, Maximum; VAI, Visceral Adiposity Index; HSI, Hepatic Steatosis Index; LAP, Lipid Accumulation Product; TyG, Triglyceride-Glucose Index; TyG-BMI, Triglyceride-Glucose Body Mass Index; TyG-WC, Triglyceride-Glucose Waist Circumference Index; TyG-WHtR, Triglyceride-Glucose Waist-to-Height Ratio; AIP, Atherogenic Index of Plasma; CMI, Cardiometabolic Index; T-C/HDL, Total Cholesterol to HDL Cholesterol Ratio; LDL/HDL, LDL Cholesterol to HDL Cholesterol Ratio; TG/HDL, Triglyceride to HDL Cholesterol Ratio.

**Table 4:** Comparison of anthropometric and body composition parameters between normal-weight and overweight/obese participants with metabolic syndrome (MetS).

	Overweight or Obese MetS (*n* = 64)				Normal-Weight MetS (*n* = 21)				
	Med	IQR	Min	Max	Med	IQR	Min	Max	*p*-Value
Age [years]	48.00	41.00–54.00	22.00	67.00	45.00	42.00–51.00	32.00	55.00	>0.05
BMR [kcal]	1579	1430–1990	1227	2542	1307	1208–1431	1172	1748	<0.001
BW [kg]	89.55	80.50–95.70	64.40	131.00	64.60	55.15–68.15	46.80	77.40	<0.001
BH [cm]	168.53	166.51–176.90	154.00	193.70	166.06	162.12–174.43	157.40	191.23	>0.05
WC [cm]	101.35	95.90–109.45	73.10	146.80	83.80	77.03–89.15	71.60	93.50	<0.001
HC [cm]	105.60	103.40–111.10	96.80	122.90	93.90	90.35–96.18	84.50	99.60	<0.001
WHR	0.97	0.92–1.02	0.68	1.19	0.88	0.85–0.92	0.82	1.00	<0.001
WHtR	0.60	0.55–0.65	0.44	0.84	0.49	0.47–0.53	0.39	0.58	<0.001
BMI [kg/m^2^]	30.08	28.21–32.53	25.35	42.53	22.30	19.68–24.64	16.90	25.00	<0.001
FFM [kg]	55.95	49.05–75.00	39.50	100.50	43.40	39.15–48.93	37.40	63.80	<0.001
FFM [%]	65.23	58.46–78.04	50.36	93.29	72.47	67.51–77.37	58.17	82.43	>0.05
SMM [kg]	30.99	27.11–43.48	21.40	59.50	23.60	21.23–26.97	20.10	36.30	<0.001
SMM [%]	36.07	32.15–44.69	27.76	54.59	39.96	36.37–41.60	30.99	46.90	>0.05
BFM [kg]	28.25	20.90–36.00	5.70	57.70	17.80	13.68–21.98	8.70	27.40	<0.001
BFM [%]	34.79	21.96–41.55	6.71	49.63	27.60	22.65–32.55	17.57	41.90	>0.05
VFA [cm^2^]	125.03	95.47–149.26	6.00	223.73	79.00	59.93–93.61	34.70	158.00	<0.001
TBW [L]	41.05	35.90–55.00	28.90	73.50	31.90	28.70–35.90	27.40	46.90	<0.001
TBW/BW [%]	47.82	42.91–57.02	37.05	68.35	53.09	49.46–56.93	42.60	60.59	>0.05
TBW/FFM [%]	73.28	73.15–73.46	72.84	73.58	73.36	73.25–73.49	73.12	73.62	>0.05
ECW [L]	15.60	13.65–20.35	11.00	26.40	12.30	10.90–13.85	10.50	17.50	<0.001
ECW/TBW [%]	37.63	37.19–38.17	35.92	39.30	38.41	37.80–38.68	37.31	39.60	<0.01
ICW [L]	25.30	22.35–34.85	17.90	47.10	19.60	17.78–22.23	16.90	29.40	<0.001
ICW/TBW [%]	62.37	61.83–62.81	60.71	64.08	61.59	61.32–62.20	60.41	62.69	<0.01

MetS, metabolic syndrome; Med, Median; IQR, Interquartile Range; Min, Minimum; Max, Maximum; BMR, Basal Metabolic Rate; BW, Body Weight; BH, Body Height; WC, Waist Circumference; HC, Hip Circumference; WHR, Waist-to-Hip Ratio; WHtR, Waist-to-Height Ratio; BMI, Body Mass Index; FFM, Fat-Free Mass; SMM, Skeletal Muscle Mass; BFM, Body Fat Mass; VFA, Visceral Fat Area; TBW, Total Body Water; ECW, Extracellular Water; ICW, Intracellular Water.

**Table 5:** Comparison of biochemical, lipoprotein, inflammatory, liver function, and hemodynamic parameters between normal-weight and overweight/obese participants with metabolic syndrome (MetS).

	Overweight or Obese MetS (*n* = 64)				Normal-Weight MetS (*n* = 21)				
	Med	IQR	Min	Max	Med	IQR	Min	Max	*p*-Value
GLU [mmol/L]	5.10	4.64–5.64	3.00	12.69	5.30	5.075–5.550	3.80	5.90	>0.05
TG [mmol/L]	1.20	0.97–1.79	0.55	3.22	1.58	0.840–2.063	0.43	2.43	>0.05
T-C [mmol/L]	5.51	4.59–6.48	3.58	8.77	5.82	4.975–6.342	3.48	7.17	>0.05
HDL-C [mmol/L]	1.41	1.16–1.64	0.40	2.48	1.56	1.29–1.85	0.96	2.38	>0.05
LDL-C [mmol/L]	3.24	2.66–3.85	1.74	7.05	3.83	2.92–4.47	1.88	5.45	<0.05
VLDL [mmol/L]	1.05	0.88–1.33	0.49	1.73	1.09	0.89–1.27	0.54	1.55	>0.05
IDL-A [mmol/L]	0.56	0.39–0.75	0.08	1.24	0.70	0.54–0.81	0.39	1.16	<0.05
IDL-B [mmol/L]	0.44	0.31–0.56	0.10	1.29	0.36	0.30–0.45	0.10	0.78	>0.05
IDL-C [mmol/L]	0.52	0.41–0.70	0.23	1.03	0.52	0.41–0.65	0.23	0.80	>0.05
LDL-1 [mmol/L]	0.88	0.72–1.09	0.28	1.73	1.06	0.97–1.17	0.67	1.42	<0.05
LDL-2 [mmol/L]	0.47	0.19–0.71	0.05	1.32	0.39	0.25–0.71	0.16	1.06	>0.05
LDL-3 [mmol/L]	0.05	0.00–0.17	0.00	0.83	0.05	0.00–0.11	0.00	0.44	>0.05
LDL-4 [mmol/L]	0.00	0.00–0.00	0.00	0.41	0.000	0.00–0.00	0.00	0.05	>0.05
LDL3-7 [mmol/L]	0.05	0.00–0.17	0.00	1.24	0.05	0.00–0.11	0.00	0.49	>0.05
Mean LDL size [nm]	27.10	26.75–27.40	25.60	27.60	27.20	26.90–27.40	26.30	27.50	>0.05
CRP [mg/L]	2.21	1.03–5.45	0.00	25.32	4.90	4.38–7.20	0.13	18.60	<0.05
UA [μmol/L]	306.00	259.64–376.00	147.00	500.00	249.00	222.25–289.00	146.00	404.00	<0.01
ALT [μkat/L]	0.35	0.26–0.47	0.14	6.58	0.21	0.18–0.32	0.10	0.45	<0.001
ALP [μkat/L]	1.27	1.04–1.58	0.73	2.07	1.06	0.90–1.57	0.62	2.10	>0.05
GGT [μkat/L]	0.36	0.30–0.53	0.16	1.21	0.28	0.20–0.36	0.16	1.31	<0.01
AST [μkat/L]	0.38	0.33–0.44	0.25	2.88	0.33	0.28–0.35	0.20	0.49	<0.001
BPs [mmHg]	132.00	124.00–140.00	106.00	155.00	124.00	113.50–139.00	106.00	147.00	>0.05
BPd [mmHg]	85.50	76.00–90.00	51.00	101.00	82.00	74.00–87.00	66.00	102.00	>0.05
Heart rate [beat/min.]	75.00	64.00–80.00	43.00	109.00	62.00	56.00–73.50	43.00	112.00	<0.05

MetS, metabolic syndrome; Med, Median; IQR, Interquartile Range; Min, Minimum; Max, Maximum; GLU, Glucose; TG, Triglycerides; T-C, Total Cholesterol; HDL-C, High-Density Lipoprotein Cholesterol; LDL-C, Low-Density Lipoprotein Cholesterol; VLDL, Very-Low-Density Lipoprotein Cholesterol; IDL-A, Intermediate-Density Lipoprotein Subfraction A; IDL-B, Intermediate-Density Lipoprotein Subfraction B; IDL-C, Intermediate-Density Lipoprotein Subfraction C; LDL-1, Low-Density Lipoprotein Subfraction 1; LDL-2, Low-Density Lipoprotein Subfraction 2; LDL-3, Low-Density Lipoprotein Subfraction 3; LDL-4, Low-Density Lipoprotein Subfraction 4; LDL3-7, Low-Density Lipoprotein Subfractions 3–7; CRP, C-Reactive Protein; UA, Uric Acid; ALT, Alanine Aminotransferase; ALP, Alkaline Phosphatase; GGT, Gamma-Glutamyl Transferase; AST, Aspartate Aminotransferase; BPs, Systolic Blood Pressure; BPd, Diastolic Blood Pressure.

**Table 6:** Comparison of metabolic, atherogenic, and cardiometabolic indices between normal-weight and overweight/obese participants with metabolic syndrome (MetS).

	Overweight or Obese MetS (*n* = 64)				Normal-Weight MetS (*n* = 21)				
	Med	IQR	Min	Max	Med	IQR	Min	Max	*p*-Value
VAI	1.63	1.15–2.32	0.51	4.40	1.71	1.05–2.72	0.54	4.39	>0.05
HSI	38.44	35.63–41.89	32.26	62.30	30.55	27.34–32.82	23.47	34.35	<0.001
LAP [cm × mmol/L]	50.32	34.33–83.57	7.37	179.70	32.44	19.41–55.64	6.84	77.04	<0.01
TyG	8.52	8.23–8.94	7.69	9.55	8.83	8.10–9.06	7.58	9.26	>0.05
TyG-BMI [kg/m^2^]	252.58	236.13–282.98	208.08	374.79	188.50	165.35–221.29	128.06	226.79	<0.001
TyG-WC [cm]	868.26	806.96–957.69	561.80	1293.67	707.54	650.17–795.98	559.98	847.60	<0.001
TyG-WHtR	5.08	4.67–5.69	3.37	7.37	4.29	3.96–4.70	2.93	5.23	<0.001
AIP	−0.01	−0.18–0.12	−0.56	0.49	−0.05	−0.28–0.14	−0.56	0.31	>0.05
CMI	0.55	0.41–0.82	0.19	1.69	0.43	0.25–0.75	0.12	1.06	>0.05
T-C/HDL	4.00	3.37–4.91	2.31	10.95	3.41	2.99–4.62	2.22	5.81	>0.05
LDL/HDL	2.39	1.80–3.09	0.96	8.36	2.25	1.75–3.20	1.09	4.50	>0.05
TG/HDL	0.98	0.66–1.31	0.28	3.08	0.90	0.52–1.37	0.28	2.05	>0.05

MetS, metabolic syndrome; Med, Median; IQR, Interquartile Range; Min, Minimum; Max, Maximum; VAI, Visceral Adiposity Index; HSI, Hepatic Steatosis Index; LAP, Lipid Accumulation Product; TyG, Triglyceride-Glucose Index; TyG-BMI, Triglyceride-Glucose Body Mass Index; TyG-WC, Triglyceride-Glucose Waist Circumference Index; TyG-WHtR, Triglyceride-Glucose Waist-to-Height Ratio; AIP, Atherogenic Index of Plasma; CMI, Cardiometabolic Index; T-C/HDL, Total Cholesterol to HDL Cholesterol Ratio; LDL/HDL, LDL Cholesterol to HDL Cholesterol Ratio; TG/HDL, Triglyceride to HDL Cholesterol Ratio.

**Table 7:** Spearman correlation analysis of metabolic, atherogenic, and cardiometabolic indices with anthropometric and body composition parameters in overweight/obese participants with metabolic syndrome (MetSO).

	BMI	VAI	HSI	LAP	TyG	TyG-WC	TyG-BMI	TyG-WHtR	AIP	CMI	T-C/HDL	LDL/HDL	TG/HDL
Age	0.074	0.087	−0.020	0.325 **	0.199	0.197	0.137	0.349 **	−0.124	−0.040	−0.339 **	−0.406 **	−0.124
BMR	0.280 *	−0.173	0.275 *	−0.316 *	−0.230	−0.083	0.130	−0.334 **	0.191	0.118	0.399 **	0.332 **	0.191
BW	0.712 ***	−0.012	0.624 ***	0.121	0.002	0.433 ***	0.596 ***	0.214	0.169	0.222	0.360 **	0.262 *	0.169
BH	−0.094	−0.245	−0.049	−0.254 *	−0.222	−0.048	−0.144	−0.380 **	0.051	−0.023	0.255 *	0.200	0.051
WC	0.717 ***	0.138	0.577 ***	0.591 ***	0.243	0.879 ***	0.709 ***	0.788 ***	−0.026	0.153	0.031	−0.070	−0.026
HC	0.892 ***	0.112	0.772 ***	0.223	0.096	0.500 ***	0.792 ***	0.390 **	0.216	0.301 *	0.310 *	0.220	0.216
WHR	0.495 ***	0.180	0.422 ***	0.641 ***	0.282 *	0.853 ***	0.534 ***	0.813 ***	−0.056	0.124	−0.047	−0.132	−0.056
FFM	0.280 *	−0.173	0.273 *	−0.315 *	−0.230	−0.083	0.130	−0.334 **	0.191	0.118	0.399 **	0.331 **	0.191
FFM	−0.295 *	−0.202	−0.217	−0.577 ***	−0.305 *	−0.565 ***	−0.382 **	−0.732 ***	0.198	0.023	0.355 **	0.327 **	0.198
SMM	0.268 *	−0.181	0.267 *	−0.335 **	−0.246	−0.104	0.111	−0.350 **	0.184	0.109	0.391 **	0.327 **	0.184
SMM	−0.250 *	−0.211	−0.174	−0.569 ***	−0.316 *	−0.536 ***	−0.347 **	−0.710 ***	0.193	0.025	0.357 **	0.320 *	0.193
BFM	0.540 ***	0.220	0.424 ***	0.653 ***	0.326 **	0.755 ***	0.599 ***	0.862 ***	−0.133	0.064	−0.232	−0.252 *	−0.133
BFM	0.294 *	0.202	0.216	0.577 ***	0.306 *	0.565 ***	0.382 **	0.731 ***	−0.197	−0.023	−0.356 **	−0.329 **	−0.197
VFA	0.468 ***	0.201	0.357 **	0.629 ***	0.322 **	0.743 ***	0.539 ***	0.840 ***	−0.139	0.044	−0.198	−0.171	−0.139
TBW	0.283 *	−0.169	0.280 *	−0.312 *	−0.226	−0.079	0.133	−0.328 **	0.193	0.122	0.399 **	0.329 **	0.193
ECW	0.293 *	−0.166	0.287 *	−0.296 *	−0.221	−0.063	0.139	−0.313 *	0.195	0.126	0.397 **	0.325 **	0.195
ICW	0.269 *	−0.178	0.270 *	−0.333 **	−0.244	−0.102	0.113	−0.348 **	0.187	0.111	0.391 **	0.327	0.187

BMR, Basal Metabolic Rate; BW, Body Weight; BH, Body Height; WC, Waist Circumference; HC, Hip Circumference; WHR, Waist-to-Hip Ratio; WHtR, Waist-to-Height Ratio; FFM, Fat-Free Mass; SMM, Skeletal Muscle Mass; BFM, Body Fat Mass; VFA, Visceral Fat Area; TBW, Total Body Water; ECW, Extracellular Water; ICW, Intracellular Water; BMI, Body Mass Index; VAI, Visceral Adiposity Index; HSI, Hepatic Steatosis Index; LAP, Lipid Accumulation Product; TyG, Triglyceride-Glucose Index; TyG-BMI, Triglyceride-Glucose Body Mass Index; TyG-WC, Triglyceride-Glucose Waist Circumference Index; TyG-WHtR, Triglyceride-Glucose Waist-to-Height Ratio; AIP, Atherogenic Index of Plasma; CMI, Cardiometabolic Index; T-C/HDL, Total Cholesterol to HDL Cholesterol Ratio; LDL/HDL, LDL Cholesterol to HDL Cholesterol Ratio; TG/HDL, Triglyceride to HDL Cholesterol Ratio. The asterisks indicate the levels of statistical significance: * *p* < 0.05, ** *p* < 0.01, and *** *p* < 0.001.

**Table 8:** Spearman correlation analysis of metabolic, atherogenic, and cardiometabolic indices with biochemical, lipoprotein, inflammatory, liver function, and hemodynamic parameters in overweight/obese participants with metabolic syndrome (MetSO).

	BMI	VAI	HSI	LAP	TyG	TyG-WC	TyG-BMI	TyG-WHtR	AIP	CMI	T-C/HDL	LDL/HDL	TG/HDL
GLU	0.207	0.164	0.106	0.255 *	0.494 ***	0.300 *	0.383 **	0.367 **	0.045	0.110	0.051	0.096	0.045
TG	0.045	0.837 ***	0.063	0.878 ***	0.936 ***	0.587 ***	0.440 ***	0.598 ***	0.708 ***	0.767 ***	0.250 *	0.106	0.708 ***
T-C	0.144	0.232	0.054	0.583 ***	0.483 ***	0.542 ***	0.340 **	0.541 ***	0.078	0.166	0.288 *	0.263 *	0.078
HDL	−0.049	−0.239	−0.097	0.271 *	0.088	0.224	0.015	0.330 **	−0.536 ***	−0.447 ***	−0.630 ***	−0.575 ***	−0.536 ***
LDL	0.090	0.189	−0.009	0.334 **	0.324 **	0.320 *	0.224	0.317 *	0.110	0.160	0.474 ***	0.601 ***	0.110
VLDL	0.051	0.317 *	0.107	0.475 ***	0.497 ***	0.381 **	0.252 *	0.295 *	0.349 **	0.374 **	0.425 ***	0.288 *	0.349 **
IDL-A	−0.081	−0.284 *	−0.221	0.121	−0.063	0.120	−0.064	0.256 *	−0.575 ***	−0.486 ***	−0.367 **	−0.194	−0.575 ***
IDL-B	0.055	0.113	0.043	0.481 ***	0.351 **	0.394 **	0.211	0.471 ***	−0.081	0.011	−0.022	−0.027	−0.081
IDL-C	0.035	0.266 *	0.015	0.575 ***	0.547 ***	0.501 ***	0.293 *	0.497 ***	0.145	0.215	0.186	0.124	0.145
LDL-1	0.020	−0.039	−0.166	0.127	0.103	0.159	0.075	0.225	−0.170	−0.116	0.202	0.318*	−0.170
LDL-2	0.249 *	0.514 ***	0.228	0.345 **	0.382 **	0.287 *	0.351 **	0.221	0.628 ***	0.632 ***	0.792 ***	0.640 ***	0.628 ***
LDL-3	0.253 *	0.516 ***	0.280 *	0.296 *	0.314 *	0.262 *	0.327 **	0.168	0.656 ***	0.651 ***	0.816 ***	0.688 ***	0.656 ***
LDL-4	0.204	0.321 **	0.264 *	0.072	0.104	0.069	0.204	−0.031	0.462 ***	0.431 ***	0.553 ***	0.489 ***	0.462 ***
LDL3-7	0.251 *	0.516 ***	0.279 *	0.289 *	0.310 *	0.255 *	0.324 **	0.161	0.657 ***	0.651 ***	0.818 ***	0.691 ***	0.657 ***
LDL size	−0.234	−0.526 ***	−0.296 *	−0.286 *	−0.333 **	−0.241	−0.318 *	−0.120	−0.697 ***	−0.683 ***	−0.753 ***	−0.561 ***	−0.697 ***
CRP	0	0.214	−0.051	0.291 *	0.259 *	0.191	0.091	0.372 **	−0.058	−0.023	−0.175	−0.073	−0.058
UA	0.333 **	0.301 *	0.245	0.409 ***	0.361 **	0.456 ***	0.414 ***	0.313 *	0.330 **	0.376 **	0.294 *	0.165	0.330 **
ALT	0.303 *	0.103	0.558 ***	−0.061	−0.011	0.052	0.244	−0.068	0.382 **	0.358 **	0.455 ***	0.325 **	0.382 **
ALP	0.123	−0.049	0.122	−0.104	−0.086	0.008	0.122	−0.084	0.092	0.091	0.239	0.292 *	0.092
GGT	0.320 **	0.296 *	0.445 ***	0.291 *	0.247 *	0.340 **	0.400 **	0.305 *	0.416 ***	0.456 ***	0.404 ***	0.304 *	0.416 ***
AST	0.169	−0.007	0.071	−0.216	−0.122	−0.164	0.086	−0.222	0.226	0.157	0.417 ***	0.373 **	0.226
BPs	−0.039	−0.231	−0.013	−0.166	−0.126	−0.083	−0.048	−0.148	−0.156	−0.167	−0.215	−0.268	−0.156
BPd	0.024	−0.058	0.028	0.224	0.074	0.263 *	0.085	0.250 *	−0.233	−0.171	−0.313 *	−0.325 **	−0.233
Heart rate	0.236	0.095	0.201	0.158	0.086	0.288 *	0.251 *	0.210	0.034	0.093	−0.047	−0.052	0.034

GLU, Glucose; TG, Triglycerides; T-C, Total Cholesterol; HDL, High-Density Lipoprotein Cholesterol; LDL, Low-Density Lipoprotein Cholesterol; VLDL, Very-Low-Density Lipoprotein Cholesterol; IDL-A, Intermediate-Density Lipoprotein Subfraction A; IDL-B, Intermediate-Density Lipoprotein Subfraction B; IDL-C, Intermediate-Density Lipoprotein Subfraction C; LDL-1, Low-Density Lipoprotein Subfraction 1; LDL-2, Low-Density Lipoprotein Subfraction 2; LDL-3, Low-Density Lipoprotein Subfraction 3; LDL-4, Low-Density Lipoprotein Subfraction 4; LDL3-7, Low-Density Lipoprotein Subfractions 3–7; CRP, C-Reactive Protein; UA, Uric Acid; ALT, Alanine Aminotransferase; ALP, Alkaline Phosphatase; GGT, Gamma-Glutamyl Transferase; AST, Aspartate Aminotransferase; BPs, Systolic Blood Pressure; BPd, Diastolic Blood Pressure; BMI, Body Mass Index; VAI, Visceral Adiposity Index; HSI, Hepatic Steatosis Index;LAP, Lipid Accumulation Product; TyG, Triglyceride-Glucose Index; TyG-BMI, Triglyceride-Glucose Body Mass Index; TyG-WC, Triglyceride-Glucose Waist Circumference Index; TyG-WHtR, Triglyceride-Glucose Waist-to-Height Ratio; AIP, Atherogenic Index of Plasma; CMI, Cardiometabolic Index; T-C/HDL, Total Cholesterol to HDL Cholesterol Ratio; LDL/HDL, LDL Cholesterol to HDL Cholesterol Ratio; TG/HDL, Triglyceride to HDL Cholesterol Ratio. The asterisks indicate the levels of statistical significance: * *p* < 0.05, ** *p* < 0.01, and *** *p* < 0.001.

**Table 9:** Spearman correlation analysis of metabolic, atherogenic, and cardiometabolic indices with anthropometric and body composition parameters in normal-weight participants with metabolic syndrome (MetSnO).

	BMI	VAI	HSI	LAP	TyG	TyG-WC	TyG-BMI	TyG-WHtR	AIP	CMI	T-C/HDL	LDL/HDL	TG/HDL
Age	0.036	0.115	0.166	0.202	0.052	0.290	0.061	0.191	−0.002	0.018	0.059	0.040	−0.002
BMR	0.203	0.010	0.325	−0.036	−0.135	0.046	0.092	−0.128	0.121	0.014	0.199	0.184	0.121
BW	0.796 ***	0.416	0.790 ***	0.478 *	0.158	0.651 **	0.677 ***	0.497 *	0.482 *	0.445 *	0.553 **	0.451 *	0.482 *
BH	−0.016	−0.190	0.069	−0.201	−0.352	−0.095	−0.203	−0.288	−0.126	−0.206	0.035	0.030	−0.126
WC	0.831 ***	0.529 *	0.783 ***	0.702 ***	0.242	0.882 ***	0.739 ***	0.798 ***	0.483 *	0.553 *	0.578 *	0.452	0.483 *
HC	0.906 ***	0.426	0.838 ***	0.509 *	0.221	0.674 ***	0.795 ***	0.584 **	0.496 *	0.487 *	0.555 **	0.454 *	0.496 *
WHR	0.635 **	0.488 *	0.626 **	0.654 **	0.241	0.790 ***	0.581 **	0.743 ***	0.381	0.472 *	0.515 *	0.406	0.381
FFM	0.219	0.038	0.331	0.003	−0.110	0.082	0.119	−0.095	0.149	0.047	0.191	0.168	0.149
FFM	−0.592 **	−0.277	−0.460 *	−0.451 *	−0.130	−0.596**	−0.555 **	−0.658 **	−0.131	−0.262	−0.335	−0.238	−0.131
SMM	0.206	0.084	0.332	0.031	−0.038	0.084	0.132	−0.081	0.196	0.084	0.218	0.209	0.196
SMM	−0.545 *	−0.168	−0.386	−0.347	−0.031	−0.510 *	−0.484 *	−0.573 **	−0.027	−0.158	−0.252	−0.160	−0.027
BFM	0.789 ***	0.390	0.648 **	0.577 **	0.160	0.749 ***	0.706 ***	0.739 ***	0.273	0.380	0.425	0.296	0.273
BFM	0.592 **	0.277	0.460 *	0.451 *	0.130	0.596 **	0.555 **	0.658 **	0.131	0.262	0.335	0.238	0.131
VFA	0.759 ***	0.383	0.636 **	0.562 **	0.129	0.748 ***	0.668 ***	0.725 ***	0.262	0.362	0.417	0.282	0.262
TBW	0.204	0.030	0.322	−0.010	−0.111	0.068	0.106	−0.110	0.143	0.038	0.184	0.164	0.143
ECW	0.187	−0.050	0.276	−0.070	−0.205	0.032	0.062	−0.145	0.058	−0.044	0.141	0.131	0.058
ICW	0.202	0.078	0.331	0.022	−0.044	0.077	0.127	−0.088	0.191	0.078	0.217	0.209	0.191

BMR, Basal Metabolic Rate; BW, Body Weight; BH, Body Height; WC, Waist Circumference; HC, Hip Circumference; WHR, Waist-to-Hip Ratio; WHtR, Waist-to-Height Ratio; FFM, Fat-Free Mass; SMM, Skeletal Muscle Mass; BFM, Body Fat Mass; VFA, Visceral Fat Area; TBW, Total Body Water; ECW, Extracellular Water; ICW, Intracellular Water; BMI, Body Mass Index; VAI, Visceral Adiposity Index; HSI, Hepatic Steatosis Index; LAP, Lipid Accumulation Product; TyG, Triglyceride-Glucose Index; TyG-BMI, Triglyceride-Glucose Body Mass Index; TyG-WC, Triglyceride-Glucose Waist Circumference Index; TyG-WHtR, Triglyceride-Glucose Waist-to-Height Ratio; AIP, Atherogenic Index of Plasma; CMI, Cardiometabolic Index; T-C/HDL, Total Cholesterol to HDL Cholesterol Ratio; LDL/HDL, LDL Cholesterol to HDL Cholesterol Ratio; TG/HDL, Triglyceride to HDL Cholesterol Ratio. The asterisks indicate the levels of statistical significance: * *p* < 0.05, ** *p* < 0.01, and *** *p* < 0.001.

**Table 10:** Spearman correlation analysis of metabolic, atherogenic, and cardiometabolic indices with biochemical, lipoprotein, inflammatory, liver function, and hemodynamic parameters in normal-weight participants with metabolic syndrome (MetSnO).

	BMI	VAI	HSI	LAP	TyG	TyG-WC	TyG-BMI	TyG-WHtR	AIP	CMI	T-C/HDL	LDL/HDL	TG/HDL
GLU	−0.122	−0.029	0.053	−0.204	−0.008	−0.181	−0.047	−0.14	0.035	−0.056	0.335	0.471 *	0.035
TG	0.340	0.847 ***	0.434 *	0.873 ***	0.968 ***	0.690 ***	0.613 **	0.735 ***	0.814 ***	0.839 ***	0.360	0.349	0.814 ***
T-C	0.470 *	0.498 *	0.624 **	0.669 ***	0.627 **	0.652 **	0.659 **	0.711 ***	0.416	0.454 *	0.464 *	0.463 *	0.416
HDL	−0.294	−0.420	−0.352	−0.174	0.014	−0.223	−0.230	−0.121	−0.476 *	−0.415	−0.741 ***	−0.688 ***	−0.476 *
LDL	0.446 *	0.596 **	0.629 **	0.616 **	0.564 **	0.610 **	0.625 **	0.645 **	0.538 *	0.553 **	0.769 ***	0.825 ***	0.538 *
VLDL	0.595 **	0.766 ***	0.700 ***	0.913 ***	0.868 ***	0.835 ***	0.813 ***	0.897 ***	0.744 ***	0.785 ***	0.491 *	0.466 *	0.744 ***
IDL-A	−0.001	−0.150	0.117	−0.040	−0.023	0.043	0.083	0.154	−0.216	−0.177	0.043	0.025	−0.216
IDL-B	0.541 *	0.550 **	0.623 **	0.554 **	0.354	0.647	0.621	0.650	0.455	0.487	0.669	0.618	0.455
IDL-C	0.367	0.397	0.539*	0.465 *	0.448 *	0.493 *	0.502 *	0.534 *	0.320	0.331	0.554 **	0.549 **	0.320
LDL-1	−0.045	−0.148	−0.011	−0.005	0.079	0.036	0.040	0.114	−0.231	−0.200	−0.007	0.106	−0.231
LDL-2	0.441 *	0.602 **	0.438 *	0.646 **	0.521 *	0.576 **	0.489 *	0.542 *	0.566 **	0.586 **	0.605 **	0.656 **	0.566 **
LDL-3	0.560 **	0.501 *	0.558 **	0.532 *	0.275	0.556 **	0.512 *	0.489 *	0.507 *	0.523 *	0.717 ***	0.712 ***	0.507 *
LDL-4	0.296	0.429	0.348	0.455 *	0.295	0.455 *	0.295	0.402	0.375	0.429	0.375	0.375	0.375
LDL3-7	0.560 **	0.501 *	0.558 **	0.532 *	0.275	0.556 **	0.512 *	0.489 *	0.507 *	0.523 *	0.717 ***	0.712 ***	0.507 *
LDL size	−0.444 *	−0.582 **	−0.423	−0.583 **	−0.418	−0.517 *	−0.442 *	−0.459 *	−0.581 **	−0.588 **	−0.611 **	−0.614 **	−0.581 **
CRP	0.155	0.530 *	0.184	0.445 *	0.444 *	0.372	0.300	0.418	0.511 *	0.561 **	0.442 *	0.522 *	0.511 *
UA	0.578 **	0.503 *	0.761 ***	0.513 *	0.287	0.605 **	0.545 *	0.551 **	0.544 *	0.536 *	0.734 ***	0.612 **	0.544 *
ALT	0.280	0.156	0.57100	0.221	0.254	0.292	0.297	0.254	0.236	0.186	0.346	0.382	0.236
ALP	0.549 **	0.314	0.573 **	0.422	0.108	0.573 **	0.446 *	0.475 *	0.352	0.371	0.395	0.385	0.352
GGT	0.423	0.383	0.620 **	0.423	0.279	0.510 *	0.476 *	0.473 *	0.411	0.392	0.556 ***	0.578 ***	0.411
AST	−0.006	−0.134	0.165	−0.061	0.053	−0.006	−0.012	−0.050	−0.081	−0.126	−0.047	0.052	−0.081
BPs	−0.557 **	−0.653 **	−0.489 *	−0.656 **	−0.409	−0.696 ***	−0.548 *	−0.607 **	−0.582 **	−0.591 **	−0.360	−0.185	−0.582 **
BPd	−0.073	−0.433	−0.073	−0.359	−0.500 *	−0.255	−0.266	−0.236	−0.515 *	−0.475 *	−0.006	−0.069	−0.515 *
Heart rate	0.229	−0.071	0.262	0.045	−0.218	0.195	0.053	0.091	−0.110	−0.123	0.228	0.164	−0.110

GLU, Glucose; TG, Triglycerides; T-C, Total Cholesterol; HDL, High-Density Lipoprotein Cholesterol; LDL, Low-Density Lipoprotein Cholesterol; VLDL, Very-Low-Density Lipoprotein Cholesterol; IDL-A, Intermediate-Density Lipoprotein Subfraction A; IDL-B, Intermediate-Density Lipoprotein Subfraction B; IDL-C, Intermediate-Density Lipoprotein Subfraction C; LDL-1, Low-Density Lipoprotein Subfraction 1; LDL-2, Low-Density Lipoprotein Subfraction 2; LDL-3, Low-Density Lipoprotein Subfraction 3; LDL-4, Low-Density Lipoprotein Subfraction 4; LDL3-7, Low-Density Lipoprotein Subfractions 3–7; CRP, C-Reactive Protein; UA, Uric Acid; ALT, Alanine Aminotransferase; ALP, Alkaline Phosphatase; GGT, Gamma-Glutamyl Transferase; AST, Aspartate Aminotransferase; BPs, Systolic Blood Pressure; BPd, Diastolic Blood Pressure; BMI, Body Mass Index; VAI, Visceral Adiposity Index; HSI, Hepatic Steatosis Index; LAP, Lipid Accumulation Product; TyG, Triglyceride-Glucose Index; TyG-BMI, Triglyceride-Glucose Body Mass Index; TyG-WC, Triglyceride-Glucose Waist Circumference Index; TyG-WHtR, Triglyceride-Glucose Waist-to-Height Ratio; AIP, Atherogenic Index of Plasma; CMI, Cardiometabolic Index; T-C/HDL, Total Cholesterol to HDL Cholesterol Ratio; LDL/HDL, LDL Cholesterol to HDL Cholesterol Ratio; TG/HDL, Triglyceride to HDL Cholesterol Ratio. The asterisks indicate the levels of statistical significance: * *p* < 0.05, ** *p* < 0.01, and *** *p* < 0.001.

## Data Availability

Data are contained within the article.

## Abbreviations

The following abbreviations are used in this manuscript:

AIP	Atherogenic Index of Plasma
ALP	Alkaline Phosphatase
ALT	Alanine Aminotransferase
AST	Aspartate Aminotransferase
BFM	Body Fat Mass
BH	Body Height
BMI	Body Mass Index
BMR	Basal Metabolic Rate
BPd	Diastolic Blood Pressure
BPs	Systolic Blood Pressure
BW	Body Weight
CMI	Cardiometabolic Index
CRP	C-Reactive Protein
ECW	Extracellular Water
ECW/TBW	Extracellular Water to Total Body Water Ratio
FFM	Fat-Free Mass
GGT	Gamma-Glutamyl Transferase
GLU	Fasting Plasma Glucose
HC	Hip Circumference
HDL	High-Density Lipoprotein Cholesterol
HSI	Hepatic Steatosis Index
ICW	Intracellular Water
ICW/TBW	Intracellular Water to Total Body Water Ratio
IDL-A, B, C	Intermediate-Density Lipoprotein Subfraction A, B, C
IQR	Interquartile Range
LAP	Lipid Accumulation Product
LDL	Low-Density Lipoprotein Cholesterol
LDL-1-7	Low-Density Lipoprotein Subfraction 1-7
Max	Maximum
Mean LDL size	Mean Low-Density Lipoprotein Particle Size
Med	Median
MetS	Metabolic Syndrome
MetSO	Metabolic Syndrome with Obesity
MetSnO	Metabolic Syndrome without Obesity
Min	Minimum
MUNW	Metabolically Unhealthy Normal-Weight
SMM	Skeletal Muscle Mass
T-C	Total Cholesterol
T-C/HDL	Total Cholesterol to High-Density Lipoprotein Cholesterol Ratio
TBW	Total Body Water
TBW/BW	Total Body Water to Body Weight Ratio
TBW/FFM	Total Body Water to Fat-Free Mass Ratio
TG	Triglycerides
TG/HDL	Triglycerides to High-Density Lipoprotein Cholesterol Ratio
TyG	Triglyceride–Glucose Index
TyG-BMI	Triglyceride–Glucose Body Mass Index
TyG-WC	Triglyceride–Glucose Waist Circumference Index
TyG-WHtR	Triglyceride–Glucose Waist-to-Height Ratio
UA	Uric Acid
VAI	Visceral Adiposity Index
VFA	Visceral Fat Area
VLDL	Very Low-Density Lipoprotein Cholesterol
WC	Waist Circumference
WHR	Waist-to-Hip Ratio
WHtR	Waist-to-Height Ratio
